# Climate Change and Maize Yield in Iowa

**DOI:** 10.1371/journal.pone.0156083

**Published:** 2016-05-24

**Authors:** Hong Xu, Tracy E. Twine, Evan Girvetz

**Affiliations:** 1 Department of Soil, Water and Climate, University of Minnesota, Saint Paul, Minnesota, United States of America; 2 International Center for Tropical Agriculture, Nairobi, Kenya; Henan Agricultural Univerisity, CHINA

## Abstract

Climate is changing across the world, including the major maize-growing state of Iowa in the USA. To maintain crop yields, farmers will need a suite of adaptation strategies, and choice of strategy will depend on how the local to regional climate is expected to change. Here we predict how maize yield might change through the 21^st^ century as compared with late 20^th^ century yields across Iowa, USA, a region representing ideal climate and soils for maize production that contributes substantially to the global maize economy. To account for climate model uncertainty, we drive a dynamic ecosystem model with output from six climate models and two future climate forcing scenarios. Despite a wide range in the predicted amount of warming and change to summer precipitation, all simulations predict a decrease in maize yields from late 20^th^ century to middle and late 21^st^ century ranging from 15% to 50%. Linear regression of all models predicts a 6% state-averaged yield decrease for every 1°C increase in warm season average air temperature. When the influence of moisture stress on crop growth is removed from the model, yield decreases either remain the same or are reduced, depending on predicted changes in warm season precipitation. Our results suggest that even if maize were to receive all the water it needed, under the strongest climate forcing scenario yields will decline by 10–20% by the end of the 21^st^ century.

## Introduction

Cereal crops, including maize, are the most important food source for human consumption [1], and as global population increases, crop production must increase. It is estimated that 60% more food will be required by 2050 (compared to 2005) to meet human nutrition needs [2]. The United States (USA) is the world’s largest producer and exporter of maize (USDA ERS; http://www.ers.usda.gov/topics/crops/corn/.aspx), and although Iowa accounts for only 1.5% of the land area of the USA, it has consistently produced more maize than any other state over the past 20 years [3], with nearly 14 million acres planted in 2012. Other top crops include soybean (9.8 million acres), and hay (1.1 million acres) (http://quickstats.nass.usda.gov). Therefore, the future of maize production in Iowa and surrounding high-producing states is a concern for the global maize economy and future food security. With maize yield variability driven strongly by climate variability, predicting future maize yields relies on understanding how climate change will affect maize growth and development.

The two climate variables most important to maize yield variability are air temperature and precipitation, radiation being the third driver. Across the current maize producing regions, increases in growing season air temperature will shorten the length of time to maturity [4], while brief spikes in temperature can affect metabolic processes, leading to reduced carbon assimilation, and cause declines in pollination and grain set [5]. Aside from large-scale atmospheric processes affecting rainfall patterns, high temperatures can also lead to moisture stress in the plant as soil moisture decreases [6,7]. Iowa has become a major producer of maize because of its ideal climate and soil environment. Situated in the center of the Midwest USA and Corn Belt region, Iowa has a humid climate with mostly loam and silty clay loam soils, requiring little irrigation (only 171,656 acres were irrigated in Iowa in 2012, compared to 8.3 million acres in neighboring Nebraska [8]); however, periods of drought can negatively affect yields. While drought has occurred in this region as a result of historical climate variability, evidence from climate models suggests that the Midwest USA could see more frequent extreme events, in particular fewer precipitation events with more intense rainfall [9]. Like most locations around the globe, average air temperature has been increasing in this region for the past 100 years, and depending on the scenario of global greenhouse gas emissions, average temperature in the Midwest is expected to increase by 3 to 8°C between the end of the 20^th^ century and the end of the 21^st^ century [10,11].

By the middle of the 21^st^ century, the Midwest USA is predicted to experience 5–20 additional days with temperatures above 35°C [10]. This is important because high temperatures can decrease carbon assimilation rates [12] as well as grain yields. A study found that at temperatures above 35°C, yields declined by 9% for each 25.4 mm reduction in precipitation [11]. But even within the range of more typical growing season temperatures, there is a negative relationship between temperature and maize yield. Lobell et al. [13] calculated a 3.1% decrease in average global maize yield as a result of temperature change, and a 0.7% decrease as a result of precipitation trends during 1980–2008. In a global analysis of maize yields for 1961–2002, Lobell and Field [14] found a 8.3% decrease in maize yield for every 1°C increase in temperature, with nearly half the variance explained by changes in minimum temperature, maximum temperature, and precipitation. Similarly, within a region of the Corn Belt in the USA in which yields were negatively correlated with temperature, Lobell and Asner [15] reported a 17% decrease in maize yield for every 1°C increase in growing season temperature for 1982–1998. The high maize yields associated with cooler summers that they reported have also been corroborated with process-based ecosystem models [16]. In a field scale manipulative experiment in 2010, maize yields decreased by 14% when in-row heaters increased mean canopy air temperature to 2.64 +/- 0.33°C above ambient [17]. While little to no significant correlation between yield and precipitation has been found in the USA, this is not because precipitation is not important to maize yield. Because of the high spatial and temporal variability of precipitation and complex interactions among soil-plant-water processes, regional to global scale analyses of yield and precipitation may not produce significant relationships, and thus smaller scale analyses might be justified to evaluate relationships among yield, temperature, and precipitation. In an analysis of data from 1976–2006 over Wisconsin (a state adjacent to Iowa), Kucharik and Serbin [18] found that maize yield decreased 13% for every 1°C increase in growing season temperature, but that this could be offset by 5–10% with modest (~50 mm) increases in precipitation.

Statistical crop yield models are useful for relating changes in reported yield values to actual climate, and observed yield variability includes variables not typically simulated within models such as pests and disease [14]. In addition to these methods, dynamic process-based simulation models are useful for simulating crop response to predicted changes in environmental variables. Previous studies have used these types of models to predict impacts of future climate change on maize yield by driving them with output from global climate models (GCMs; e.g., [19]), however, as crop models and GCMs are improved, and scenarios of future global climate and land use are updated, new simulations must be performed to reflect the current state of yield predictability. Here we present results of simulations of the Agro-IBIS model driven with the latest projections of future climate. Agro-IBIS has been evaluated with field scale measurements of water use, crop growth, and phenology [20,21], and with reported maize yield across a 13-state region (including Iowa; [22]) and the Upper Mississippi River basin [23]. Grid cell values of leaf area index have been evaluated with remotely sensed measurements across the central and eastern USA [24] to quantify crop phenology. The model has been used in many studies relevant to climate change impacts, including analyses of maize water use under both present and future greenhouse gas concentrations [25,26], 20^th^ century USA trends in crop productivity [16], and impacts of changing planting dates and cultivars on yields and water use [27].

The recently released series of GCM datasets associated with the Coupled Model Intercomparison Project phase 5 (CMIP5) project provide state of the art projections of future climate using several different scenarios [28]. Along with revised emissions and land use scenarios called representative concentration pathways (RCPs), the CMIP5 simulations have many improvements over the previous CMIP3 simulations, including a finer horizontal resolution of the atmospheric component in many of the models [29].

Here we report results of a series of Agro-IBIS simulations driven with downscaled CMIP5 output used to predict maize yield in Iowa for the 21^st^ century. We use the Agro-IBIS model to separately evaluate maize response to projected temperature and precipitation changes from two RCPs. We limit our domain to the state of Iowa in order to diminish spatial variability in precipitation patterns and to predict future maize yield in a highly productive region.

## Materials and Methods

### Study Objective and Input Climate Datasets

In this study, we used the output of six GCMs from CMIP5 (CSIRO-Mk3.6.0, GISS-E2-R, IPSL-CM5a-MR, MIROC5, CCSM4, GFSL-ESM2m; S1 Table) to run three scenarios: a historic run (HISTORIC), the midrange mitigated emission scenario (RCP4.5), and the high emission scenario (RCP8.5) [29–31] with Agro-IBIS [21] to predict Iowa maize yield. We also define a Yield Loss Index (YLI) to partition effects of moisture stress from temperature stress.

We address one of the uncertainties involved with GCMs, that is, uncertainty from model bias related to model structure and climate sensitivity, by using output from several GCMs. Monthly values of maximum and minimum air temperature, precipitation, specific humidity, and cloud cover were downscaled and bias-corrected from their native grids to a 0.5° by 0.5° latitude/longitude grid using quantile mapping to historical climate data for the 1901–2005 period provided by the University of East Anglia Climate Research Unit (CRU; [32]). The bias correction was conducted using the R statistical program contributed package Qmap, which performs a quantile mapping using robust empirical quantiles [33]. Extrapolation beyond the range of the historical distribution was conducted using a linear interpolation scheme [34]. This is the same method of downscaling used in a previous study by the authors over a single model grid cell in Iowa [35] and allows comparison of results from this study with those of the previous study while minimizing differences between the studies. We selected these particular GCMs because they simulate historical values of temperature and precipitation that are similar in magnitude and seasonal cycle to values from the historical CRU dataset after downscaling. For more details on simulated climate from these models in Iowa, please see [35].

### Agro-IBIS Model Description

Agro-IBIS is a dynamic ecosystem model adapted from the Integrated Biosphere Simulator [36,37] to simulate land surface processes within natural and managed ecosystems. The model simulates fast response processes such as energy, water, and carbon fluxes in the vegetation canopy and soil, intermediate processes such as leaf growth, and slow response processes like soil carbon storage and turnover. Net primary productivity is simulated at each hourly time step by scaling the net effects of photosynthesis and autotrophic respiration to the canopy. For C4 species such as maize, the model uses a coupled model of photosynthesis and stomatal conductance [38,39]. Input requirements include solar radiation or cloud cover, air temperature, precipitation, humidity, wind speed, and soil texture classification for 11 soil layers from the surface to 2.5 m. In this study we use a weather generator to derive hourly input variables from the monthly GCM inputs, and we use soil texture class provided by the CONUS soil database [40].

The growth stages of crops including planting, emergence, grain fill, senescence, and harvest are simulated according to accumulated growing degree days (GDD). Planting date is determined as the date when the 10-day average air temperature is higher than 10°C and the 10-day average minimum temperature is higher than 6°C. The number of GDD required to reach maturity is a model parameter that can be fixed or can vary spatially or temporally in response to long-term changes in climate. The fraction of carbon allocated to leaves, stems, and roots changes with each growth stage and decreases to zero after peak leaf area index is reached while allocation to reproductive organs increases to one (i.e., 100%).

Simulated crops respond to changes in temperature either physiologically by changing carbon assimilation rates or phenologically by shifting the timing of the various growth stages as GDD are accumulated more or less rapidly over the growing season. Growing degree days between maize growth stages are summed using the difference between the mean of the daily maximum and minimum air temperature and the base temperature of 8°C. A maximum temperature of 30°C is imposed. Assimilation is also modified by nitrogen and water stress. Nitrogen is applied once at the beginning of the growing season and hourly canopy and soil processes determine water that is available to roots for transpiration. Changes in all of these processes contribute to changes in crop yield by changing the magnitude of assimilation or crop duration.

### Agro-IBIS Model Evaluation Using Historical Data

Before using Agro-IBIS to predict the yield response to climate change, it is important to ensure that the model is capable of simulating reasonable yield values and capturing the inter-annual variability as crops respond to variations in input climate. Kucharik [22] showed that Agro-IBIS is able to simulate realistic corn yields across a 13-state region that contains our study domain, and that the model captures interannual variability in yield as corn responds to variable climate. Here we focused our evaluation on the state of Iowa and compared our simulated yield with county level reported yield data from 1951–2005 [41]. We drove Agro-IBIS with climate data from the six GCMs used in our future scenarios, as well as with CRU data that are based on observations [32]. Agro-IBIS was run in a region encompassing 40.75° to 43.25° N latitude and -96.25° to -90.75° longitude, which covers most of the state of Iowa in the USA, at a 0.5° by 0.5° grid cell resolution. This resolution produces 72 grid cells in our domain. We combined monthly values of the model-required variables from the CRU dataset with daily anomalies of reanalyzed meteorological data from the National Centers for Environmental Prediction-National Center for Atmospheric Research (NCEP/NCAR; [42]) and used a weather generator to derive hourly values for each model time step. Nitrogen fertilizer rates were based on historic records and increase from 3.5 kg ha^-1^ in 1951 to 135 kg ha^-1^ in 1985 (a nearly linear increase of 3.8 kg ha^-1^ yr^-1^) and then continue at this rate for each successive model year. The maize cultivar was fixed at each grid cell to require 1700 GDDs to reach maturity. While actual maize cultivar will vary across our domain and possibly from field to field, we fix GDD at 1700 to use a value that is within the realistic range of values but is high enough to allow analysis of yield changes under a warming climate (i.e., farmers will likely use cultivars with a greater GDD as climate warms in order to maximize yields).

### Experimental Simulations

We performed two experimental simulations for each GCM driving dataset and each RCP scenario (2 experiments x 6 GCM datasets x 2 RCP = 24 output datasets). In the first experiment we ran the model with default settings, which includes the ability to stress vegetation from lack of moisture. In the second experiment the only change we made was to switch off the moisture stress parameter such that moisture was not a variable in the photosynthetic stress function. In Agro-IBIS, moisture stress is a scaling factor that is calculated based on the available water content in the soil and the root profile of the plant, and applied on the maximum Rubisco capacity of photosynthesis. Its value ranges from 0 (maximum stress) to 1 (no stress). We refer to the default setting scenarios as DEFAULT, and to the non-moisture stressed scenarios as NONSTRESS. Because moisture stress is essentially caused by lack of precipitation in our study’s humid domain, the difference in yield change between DEFAULT and NONSTRESS can be attributed to the change in precipitation only (i.e., not temperature, humidity, or radiation). In other words, while yield responds to the overall climate variation and stresses between the HISTORIC runs and RCP runs in the DEFAULT simulations, yield in the NONSTRESS simulations can be understood as the optimum yield that can be achieved under no moisture stress, possibly from full irrigation if necessary.

In order to quantify the extent to which precipitation deficiencies affect yield, we define a Yield Loss Index (YLI) as

$$YLI=\frac{Yield_{D-}\;Yield_{NS}}{Yield_{NS}}$$ (1)

where Yield_D_ is the yield from the DEFAULT run and Yield_NS_ is the yield from the NONSTRESS run. The index is bounded between -1 and 0 and is expected to decrease (i.e., become more negative) with time if moisture stress increases with future climate patterns. Because we calculate the YLI over several decades, the index can be thought of as a descriptive long-term climatic yield gap. It quantifies the long-term average gap between a yield optimum and a realistic yield, based solely on average moisture availability; therefore, if a location were to experience significant changes over time to precipitation patterns or other processes that affect soil moisture, we would expect the YLI to change.

All the simulations started with an accelerated spin-up to allow the soil carbon to reach near-equilibrium conditions. Then the model was run continuously from 1861–2005 with output from GCM HISTORIC runs, and from 2006–2100 for both RCP4.5 and RCP8.5 scenarios. Because our goal is to evaluate the effects of changes in temperature and precipitation on yield, we did not allow the maize to become nitrogen stressed, we used a fixed, relatively high growing-degree day cultivar (1700 GDD) to quantify how the growing period would be advanced with increasing temperature, and we held atmospheric carbon dioxide concentration at 350 ppm. We analyze simulated maize yield and other variables within three 20-year periods: 1981–2000 (referred to as HISTORIC), 2041–2060 (referred to as MID21), and 2081–2100 (referred to as LATE21).

## Results

### Agro-IBIS Simulation of Historic Maize Yields

**Fig 1:**
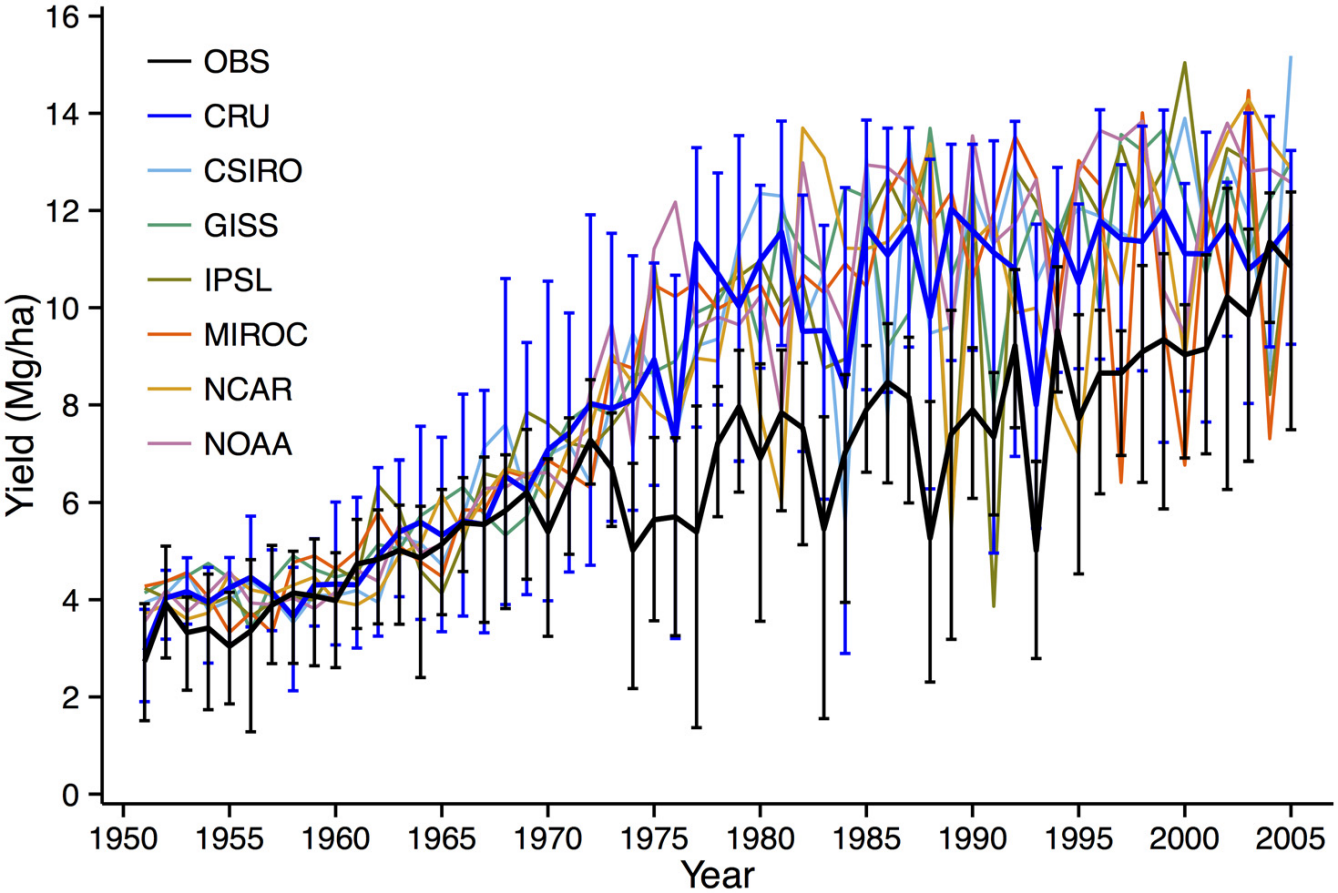
Agro-IBIS simulated maize yield (Mg ha^-1^) as driven with CRU data and output from all six GCMs for 1951–2005, and maize yield surveyed by USDA. Simulated values are averaged over all grid cells in the model domain, and county level observations are averaged over a region corresponding to the model domain. Error bars indicate the range of yield across grid cells (for model simulation) and counties (for survey).

Agro-IBIS captures the observed increase in yield between 1951 and 2005 (Fig 1). While the increase in historic yield is attributed to a combination of breeding and management practices [43], Agro-IBIS implicitly simulates this trend as a result of increasing nitrogen fertilizer application rates. Simulated yields averaged across all grid cells driven by CRU data are overestimated compared with observations between 1970 and 2000, likely a result of the model only responding to nitrogen and moisture stress but to no other stresses that can affect actual crop yields, as well as the fixed GDD required for maturity at a relatively high value. However, given the high spatial variability in yield in both simulations and observations in recent decades, data in Fig 1 provide evidence that the model captures the inter-annual variability in response to climate variation quite well (R = 0.87) because of the overlap of error bars in 54 of 55 years. Additionally, our analysis of the same observations and model simulation over a single model grid cell in Iowa showed that while the model overestimated yield in high yielding years, the model captured well the low yields (< 5 Mg ha^-1^) of low yielding years (see Fig 6a in [35]). Simulated yields from the GCM-driven runs are closer to the CRU runs than the observations. The average yield of all six models over the whole run and domain is 6.90 Mg ha^-1^, and the average observed value is 6.75 Mg ha^-1^.

### Future Scenarios of Temperature and Precipitation in Iowa from the CMIP5 Data

**Fig 2:**
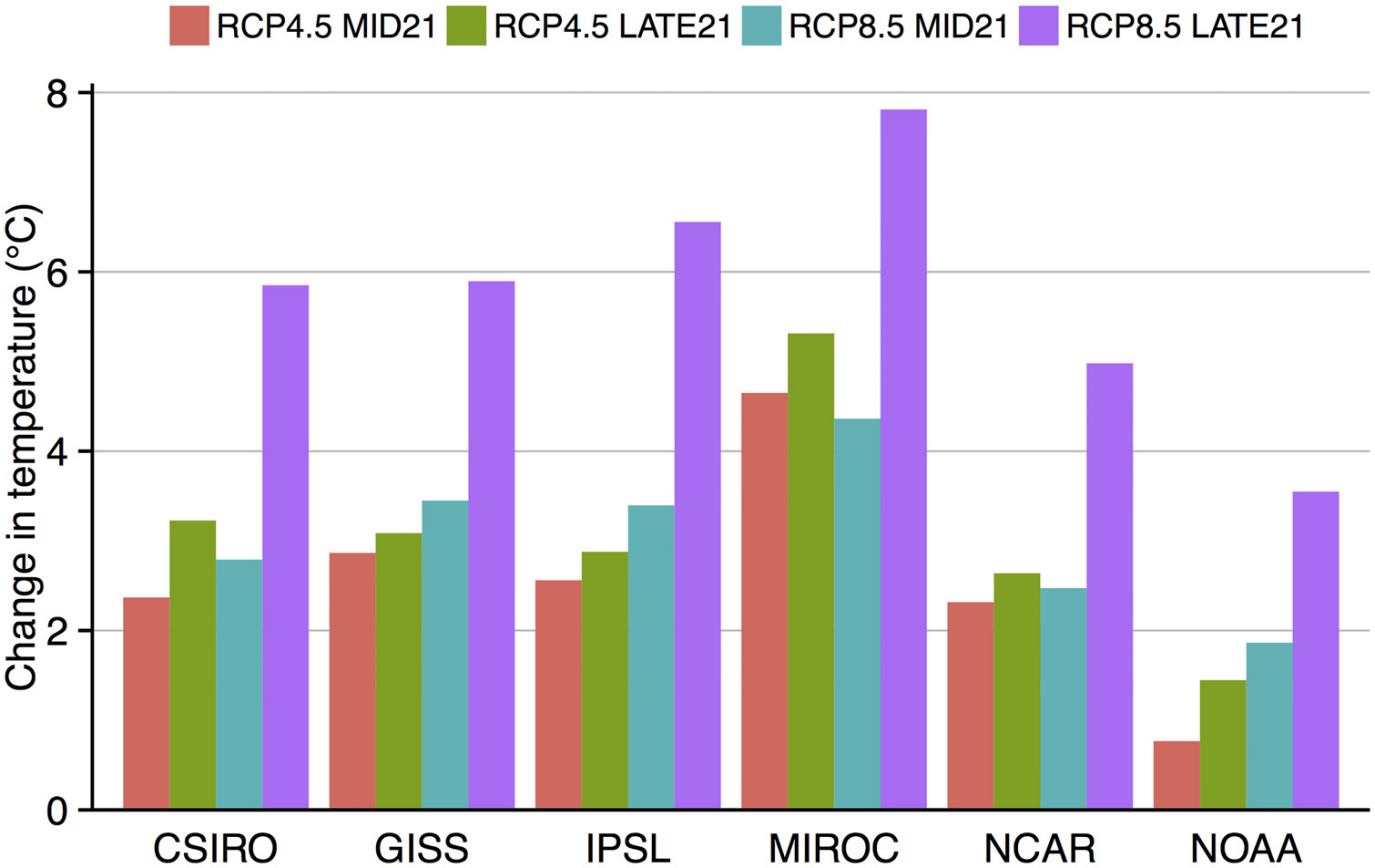
Change in the May-October average air temperature (°C) for each GCM and RCP scenario where the value shown is a difference between MID21 or LATE21 and HISTORIC.

Under both RCP scenarios and for both MID21 and LATE21, all CMIP5 models in our study predict the Iowa average growing season (May-October) temperature to increase compared to HISTORIC (Fig 2). Additional increases in average temperature occur between MID21 and LATE21, and between RCP4.5 and RCP8.5, with variations among the models as a result of differences in climate sensitivity and model structure. Under the RCP4.5 scenario, four models predict an increase in temperature for MID21 of 2–3°C, while the NOAA model predicts a smaller increase of 0.8°C and the MIROC model predicts a larger increase of 4.6°C. In all models, the additional increase in temperature by LATE21 is less than 1°C because in the RCP4.5 scenario greenhouse gas emissions have stabilized by the late 21^st^ century.

Under the RCP8.5 scenario for MID21, average May-October air temperature increases in four models by ~3°C, while the lower climate sensitivity of the NOAA model results in a 1.9°C increase and the higher climate sensitivity of the MIROC model results in a 4.4°C increase. Because the radiative forcing continues to increase throughout the 21^st^ century in RCP8.5, the increase in air temperature has almost doubled by LATE21 compared to MID21 with increases ranging from 3.5°C as predicted by the NOAA model to almost 8°C as predicted by the MIROC model.

**Fig 3:**
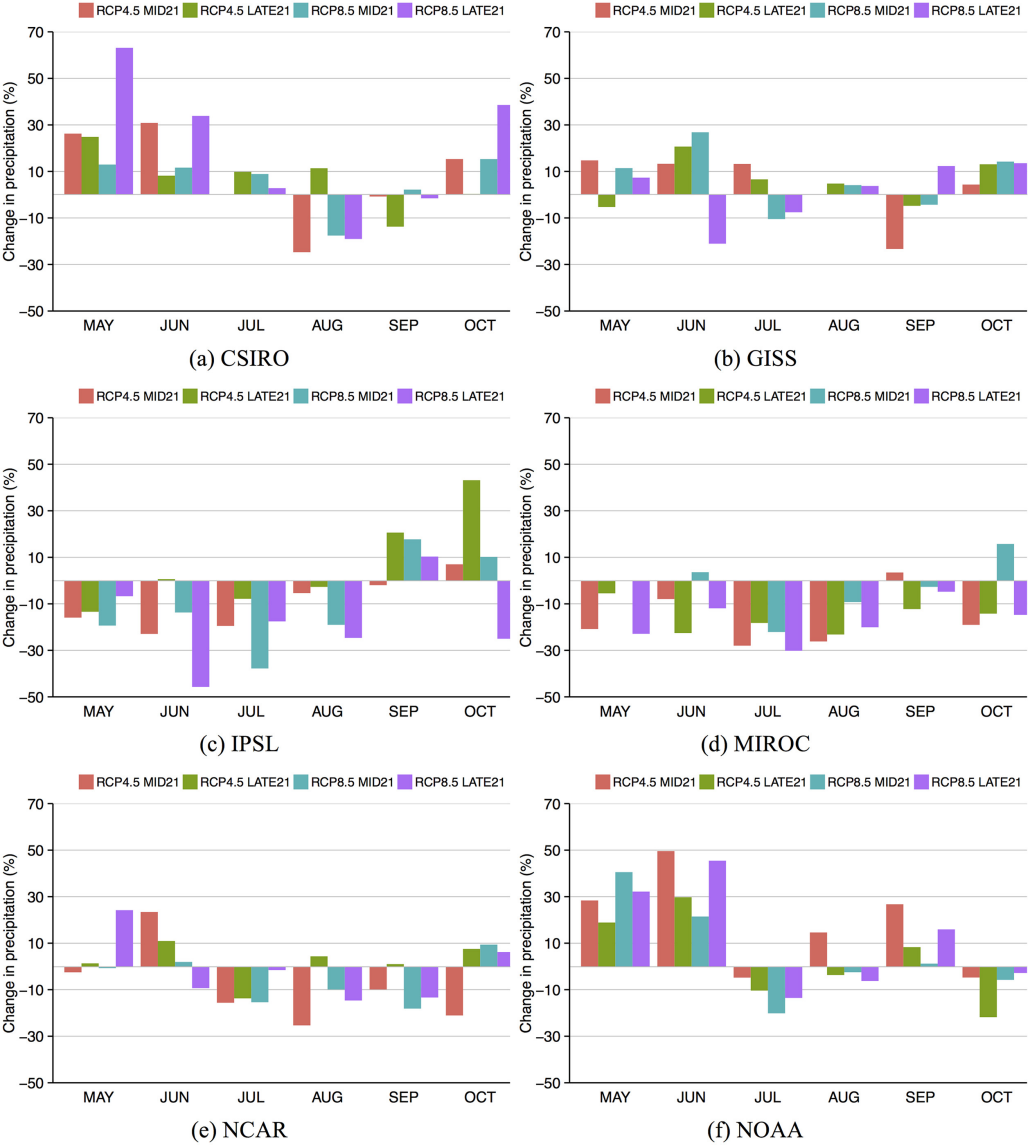
Change in monthly precipitation for May through October for each GCM and RCP scenario where the value shown is a percent difference between MID21 or LATE21 and HISTORIC.

Predictions of growing season precipitation from the GCMs are more variable than predictions of temperature, therefore we analyze these changes by month between May and October (Fig 3). While all models predict either no change or decreases in precipitation in mid-summer, changes during the early and later portions of the season vary with some models predicting more and some predicting less precipitation. The GISS and NCAR models show small to moderate increases and decreases throughout the season (mostly < 20%), while the IPSL and MIROC models show moderate decreases (mainly within 30%), and the CSIRO and NOAA models predict a wetter early season (> 20%) and then drying in mid-season (0–20%). One example of the difficulties in predicting yield response to climate variations is shown in the MIROC model results. The greater radiative forcing of RCP8.5 does not result in a greater increase in air temperature in the MIROC output compared with RCP4.5 (Fig 2) because during most months of the growing season precipitation is greater in the RCP8.5 runs than in the RCP4.5 runs (Fig 3), thereby allowing more water to cool the land surface and mitigate increased air temperature.

### Agro-IBIS Predicted Changes in Default Yield

Differences in predicted changes in yield are larger among the different model runs than across the model domain for any one scenario (S1 and S2 Figs). In other words, the predicted climate from a particular GCM and RCP scenario does not vary greatly across the state of Iowa. It is because of this homogeneous response that we summarize projections with a state-wide average value of yield in tables and figures, however, simulation results are presented for each model grid cell in some figures and shown across the entire domain for each run and RCP scenario in S1 and S2 Figs.

All six HISTORIC model runs simulate a 1981–2000 average DEFAULT (i.e., including moisture stress) yield near 11 Mg ha^-1^ (Table 1), which is close in value to the late 20^th^ century / early 21^st^ century observed value averaged over all counties (Fig 1). All statistically significant changes in maize yield (p < 0.01) by the middle and late 21^st^ century are negative. These decreases in yield range from 13–15% under RCP4.5 in both MID21 and LATE21 in the GISS model, to a ~50% decrease under RCP8.5 by LATE21 in the IPSL and MIROC models.

**Table 1:** Simulated DEFAULT (i.e., including moisture stress) maize yield (Mg ha^-1^) averaged across all 72 grid cells in domain from each HISTORIC run driven by the six GCMs and the percent change between HISTORIC and the MID21 and LATE21 runs under both RCP scenarios.

Model	Yield (Mg ha^-1^) HISTORIC	Change in yield (%)			
		RCP4.5		RCP8.5	
		MID21	LATE21	MID21	LATE21
CSIRO	11.11	-11.71	-9.42	**-17.85**	**-19.52**
GISS	11.54	**-15.28**	**-13.61**	**-19.06**	**-36.68**
IPSL	11.69	**-28.77**	-16.69	**-34.40**	**-50.08**
MIROC	11.53	**-42.16**	**-42.30**	**-30.05**	**-48.86**
NCAR	10.71	-13.91	-12.59	-16.17	**-19.01**
NOAA	11.88	1.48	-9.18	-9.94	-14.00

Bold values denote changes that are statistically significant at P<0.01 according to a Student’s t-test.

**Fig 4:**
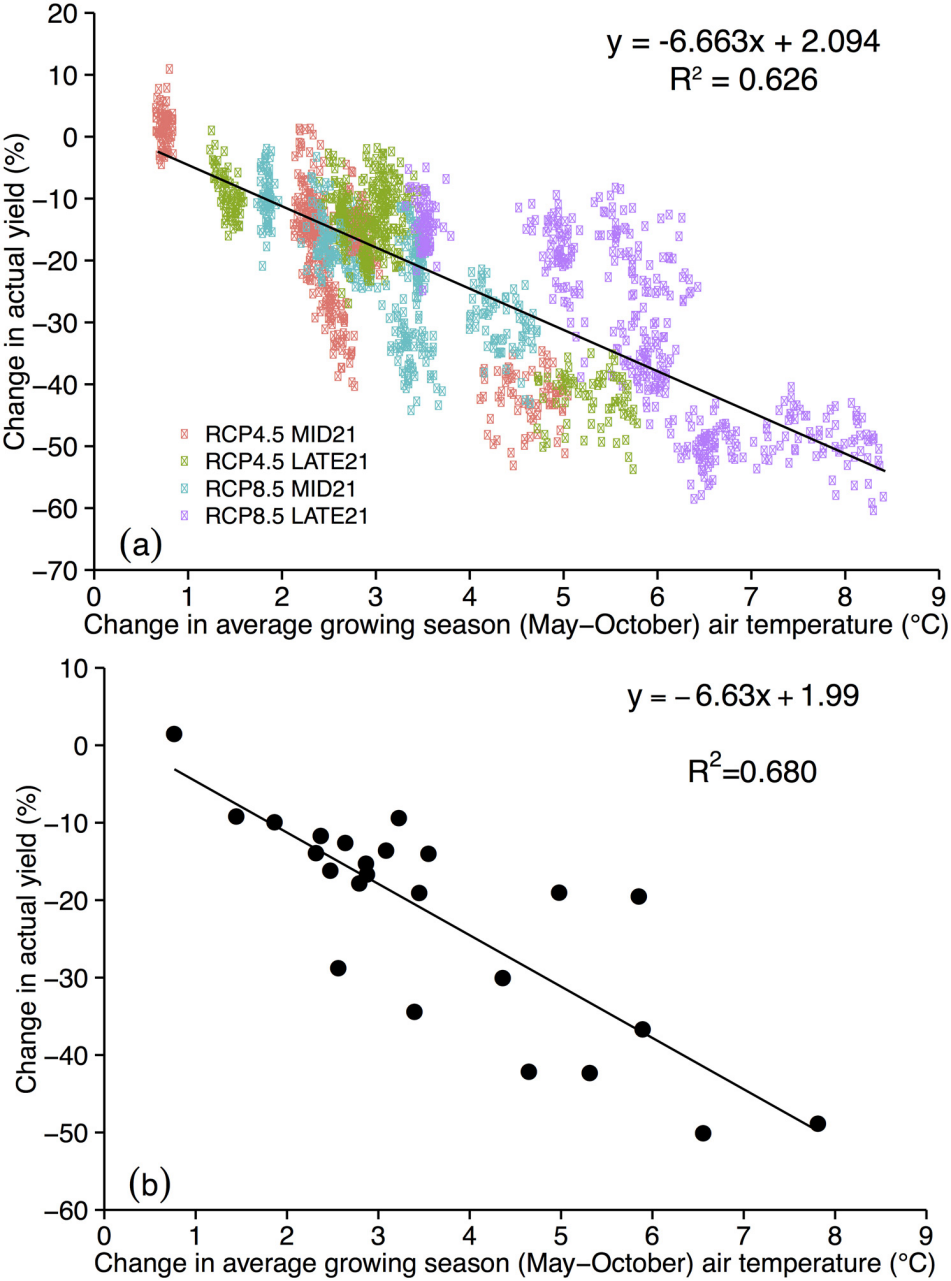
Change in DEFAULT yield (%) vs. change in May-October average air temperature (°C) for each GCM and RCP scenario where the value shown is a difference between MID21 or LATE21 and HISTORIC. Each point represents the value of a model grid cell (a) or the domain average (b).

Differences in predicted yield among model runs can be explained by the combination of changes in temperature and rainfall timing and magnitude. For example, even though GISS and IPSL show similar patterns of warming for each RCP scenario and time period (Fig 2), IPSL runs predict larger yield declines (Table 1) because of greater decreases in rainfall from May through August (Fig 3). Similarly, NCAR yield declines are not statistically significant until LATE21 under RCP8.5 because of less warming than four other models (Fig 2), and less summer drying after some early spring increases (Fig 3). CSIRO predicted yield declines are similar to the NCAR runs (Table 1) even though warming is larger in each RCP and time period (Fig 2) because of large increases in spring and early summer rainfall (Fig 3) that compensate for the large warming. Yield declines predicted by MIROC under RCP4.5 (Table 1) are larger than any other model because MIROC has greater warming than other models (Fig 2) and decreases in rainfall in all months (Fig 3). This extreme warming is also found in MIROC under RCP8.5, however, yield declines are similar to those of IPSL in MID21 and LATE21 (Table 1) because the May-June MIROC rainfall decreases are not as severe as in IPSL (Fig 3). One result of the high climate sensitivity of the MIROC model is found in yield predictions for MID21. Yield is predicted to decrease more under RCP4.5 (by 42%) than under RCP8.5 (by 30%; Table 1) because May-August rainfall decreases are larger in RCP4.5 than in RCP8.5 (Fig 3). The greater summer rainfall in RCP8.5 mitigates warming as well (as explained above), thereby mitigating yield declines. A linear regression of all grid cells (Fig 4a) and of the domain average (average of all 72 grid cells; Fig 4b) predicts that for every 1°C warming during the growing season, Iowa can expect a 6.6% reduction in maize yield.

### Agro-IBIS Predicted Changes in Non-Stressed Yield

All six HISTORIC model runs simulate a 1981–2000 average NONSTRESS yield near 14 Mg ha^-1^ (Table 2), which is 3 Mg ha^-1^ larger than the HISTORIC DEFAULT yield. Removal of moisture stress in these model runs still results in statistically significant predicted decreases in yield from five of the six models in the RCP4.5 scenario and from all six models in the RCP8.5 scenario; however, the decreases are smaller than in the DEFAULT run. For the RCP4.5 scenario, predicted decreases in yield range from ~5% in the NCAR runs to 15% in the MIROC runs. Again, as with the DEFAULT runs, the predicted changes in RCP4.5 do not vary greatly between MID21 and LATE21 because emissions stabilize near mid-century. In contrast, in the RCP8.5 scenario predicted yield decreases are amplified with time and range from 5% in the NCAR runs in MID21 to 22% by LATE21 in the MIROC runs.

**Table 2:** Simulated NONSTRESS maize yield (Mg ha^-1^) averaged across all 72 grid cells in domain from each HISTORIC run driven by the six GCMs and the percent change between HISTORIC and the MID21 and LATE21 runs under both RCP scenarios.

Model	Yield (Mg ha^-1^) HISTORIC	Change in yield (%)			
		RCP4.5		RCP8.5	
		MID21	LATE21	MID21	LATE21
CSIRO	14.02	**-5.76**	**-10.06**	**-12.33**	**-18.82**
GISS	13.98	**-8.87**	**-10.88**	**-10.99**	**-19.27**
IPSL	14.11	**-9.23**	**-7.49**	**-9.77**	**-19.50**
MIROC	14.05	**-15.40**	**-13.73**	**-9.95**	**-22.50**
NCAR	13.74	**-4.79**	**-7.58**	**-5.42**	**-14.63**
NOAA	13.96	-2.74	-2.91	**-4.84**	**-10.79**

Bold values denote changes that are statistically significant at P<0.01 according to a Student’s t-test.

One exception to the NONSTRESS runs mitigating yield loss compared with DEFAULT is the CSIRO model. By LATE21 under RCP8.5, yield declines are similar for CSIRO runs for both NONSTRESS and DEFAULT because increases in early summer rainfall in future runs offset drying in August. In other words, even though the DEFAULT model runs allow moisture stress, increased early summer rainfall did not allow this occur, and thus all yield decreases for this model and scenario are a result of temperature increase.

The predicted decreases in NONSTRESS yield are a combined effect of the change in several environmental factors, among which temperature and solar radiation are usually assumed to be the most important. Runs using output from five of the six GCMs predict a small increase in the net solar radiation in the future for both RCP scenarios (S3 Fig). These values range from <1% to ~7%. These small increases, which alone should force increases in yield, suggest that the predicted decreases in NONSTRESS yield can be mostly attributed to increases in air temperature.

Temperature can affect both photosynthesis rate and phenology. To quantify the effects on phenology, we analyzed how the growing period of maize changed in the NONSTRESS runs.

Because we set the number of GDD to maturity to 1700 in all runs, increases in average growing season temperature will accumulate GDD faster and result in earlier maturity and a shortened growing period (defined as the number of days between the planting date and physiological maturity). Model simulations from both RCP scenarios and all GCMs predict a decreased growing period for both future time periods (Table 3). Results range from 10 fewer days (NOAA model runs for RCP4.5 LATE21) to 34 fewer days (IPSL and MIROC model runs for RCP8.5 LATE21).

**Table 3:** Simulated average maize growing period length averaged across all 72 grid cells in domain from each HISTORIC NONSTRESS run driven by the six GCMs and the change in number of days between HISTORIC and the MID21 and LATE21 NONSTRESS runs under both RCP scenarios.

Model	Growing period (days) HISTORIC	Change in the growing period length			
		RCP4.5		RCP8.5	
		MID21	LATE21	MID21	LATE21
CSIRO	147	**-18**	**-22**	**-24**	**-30**
GISS	149	**-21**	**-23**	**-24**	**-32**
IPSL	147	**-23**	**-22**	**-25**	**-34**
MIROC	149	**-29**	**-30**	**-26**	**-34**
NCAR	144	**-19**	**-21**	**-19**	**-28**
NOAA	147	-5	**-10**	**-15**	**-24**

Bold values denote changes that are statistically significant at P<0.01 according to a Student’s t-test. A negative value indicates a shortening of the growing period.

**Fig 5:**
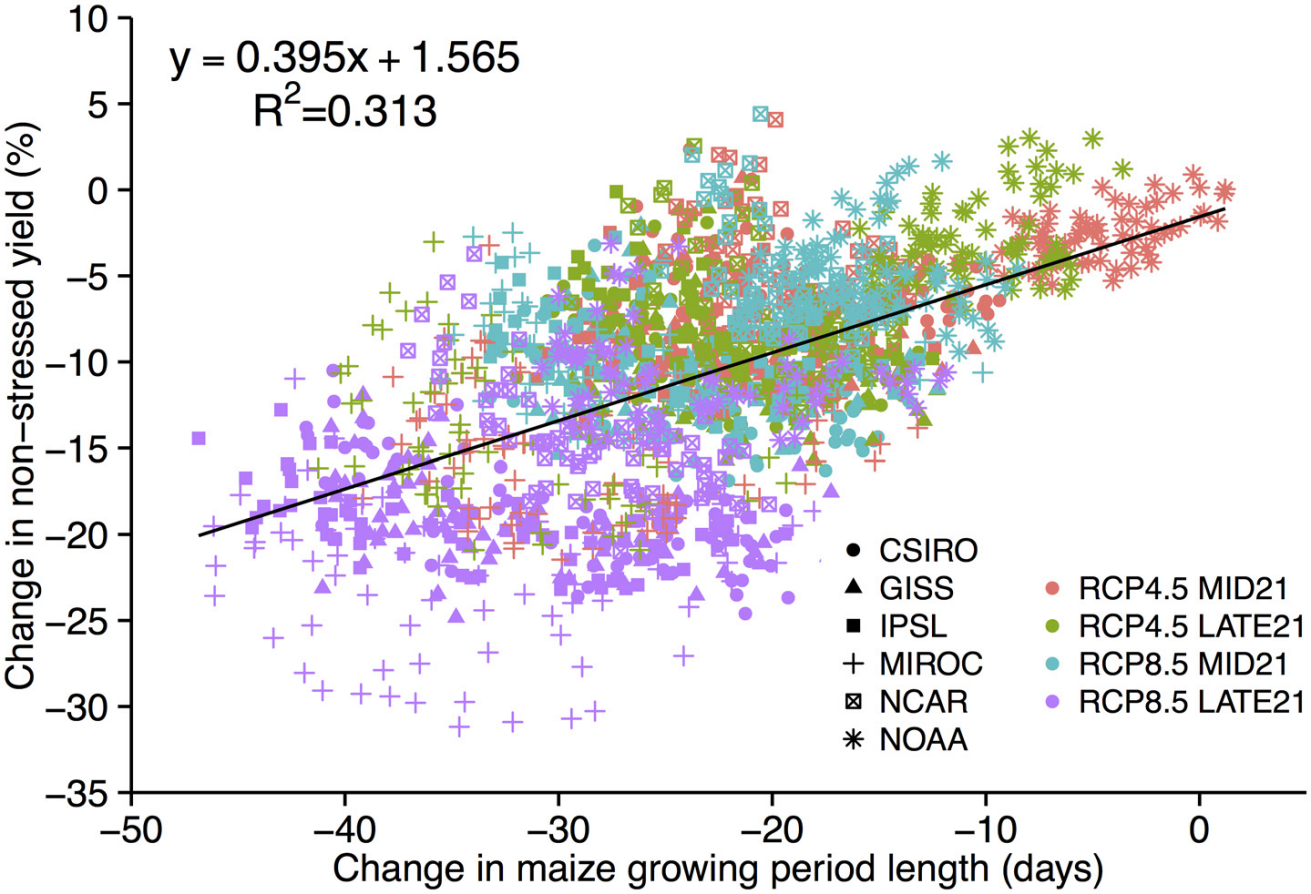
Change in NONSTRESS yield (%) vs. change in maize growing period length (days) for each GCM and RCP scenario where the value shown is a difference between MID21 or LATE21 and HISTORIC. Each point represents the value of a model grid cell.

The simulated shortened growing period and earlier maturity generally results in decreased maize yield (Fig 5). Our analysis shows that for every two days lost to growth, the yield decreases by just under 1%; however, there is great variability in this relationship (R^2^ = 0.31), especially later in the 21^st^ century with higher emissions (LATE21 and RCP8.5) as responses to increased temperature other than shortened growing period impact yield. The low scatter about the regression line at small decreases in growing period length implies that a small decrease in growing period (generally RCP4.5 and MID21) can have a robust effect on yield, while with higher emissions (RPC8.5 and LATE21) the corresponding increase in temperature plays an increasing role in affecting photosynthetic capacity. For example, the decreases in growing period length for the CSIRO, GISS, IPSL, and NCAR RCP4.5 runs are all nearly 20 days which translates to a yield decrease of 8%. This value is similar to the NONSTRESS yield declines in Table 3 implying that reduction in growing period length dominates the yield change signal. The MIROC RCP8.5 LATE21 runs predict the largest decreases in growing period length of 34 days. A 34-day decrease in growing period is equivalent to a yield decline of 13% according to the regression—this is less than the 22% yield decline in NONSTRESS MIROC runs, implying that extreme temperatures are reducing photosynthetic capacity by an additional 9% beyond decreased growing period length.

**Fig 6:**
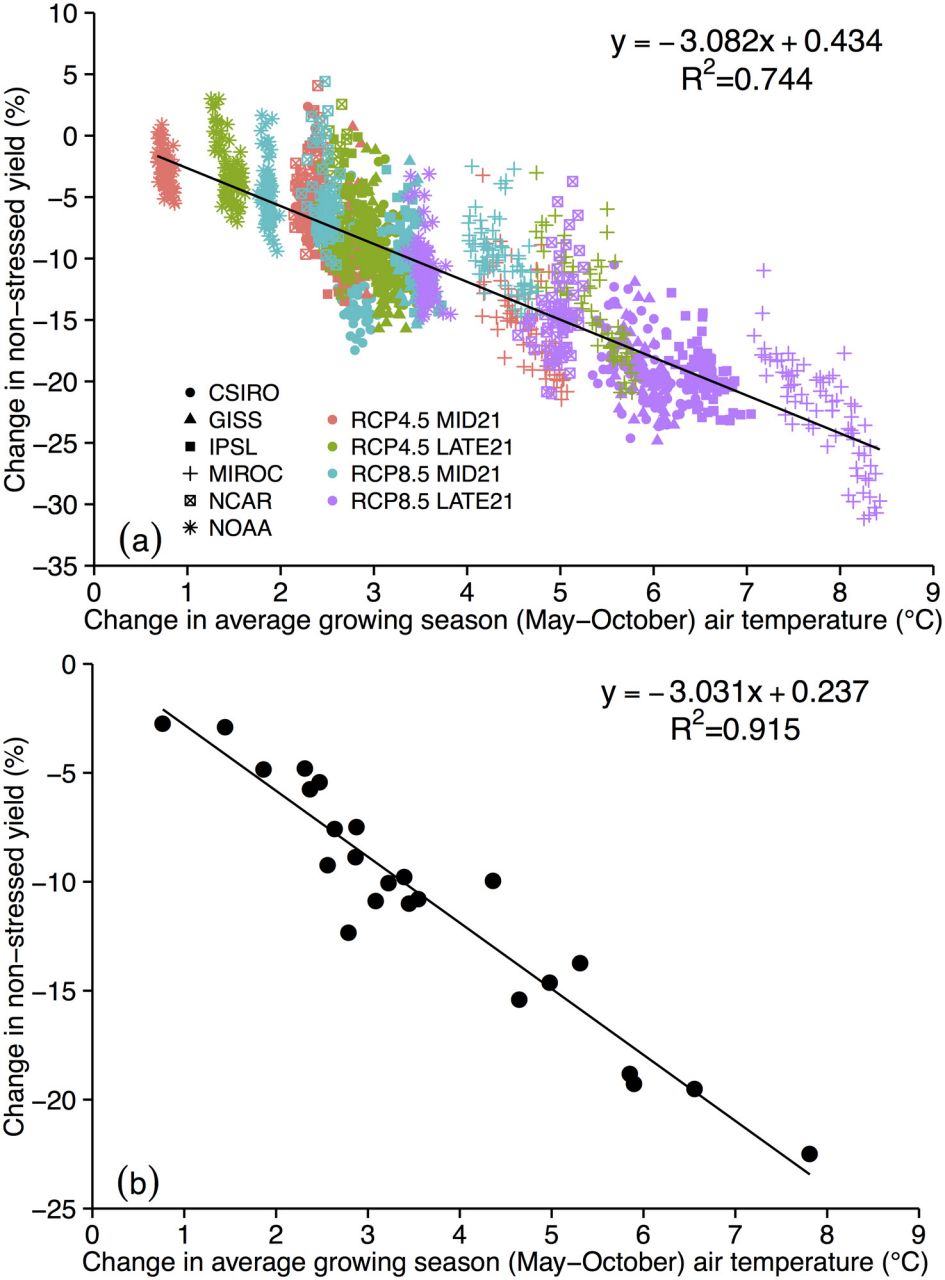
Change in NONSTRESS yield (%) vs. change in May-October average air temperature (°C) for each GCM and RCP scenario where the value shown is a difference between MID21 or LATE21 and HISTORIC. Each point represents the value of a model grid cell (a) or the domain average (b).

Our analysis finds a strong relationship (R^2^ = 0.74) between increases in growing season average air temperature and reductions in NONSTRESS yield (Fig 6a). When only domain averages are analyzed, the correlation becomes even stronger (R^2^ = 0.91; Fig 6b). The regression suggests that for every 1°C warming during the growing season, Iowa can expect a 3.0% reduction in non-moisture-stressed maize yield.

### Agro-IBIS Predicted Yield Loss Index

**Fig 7:**
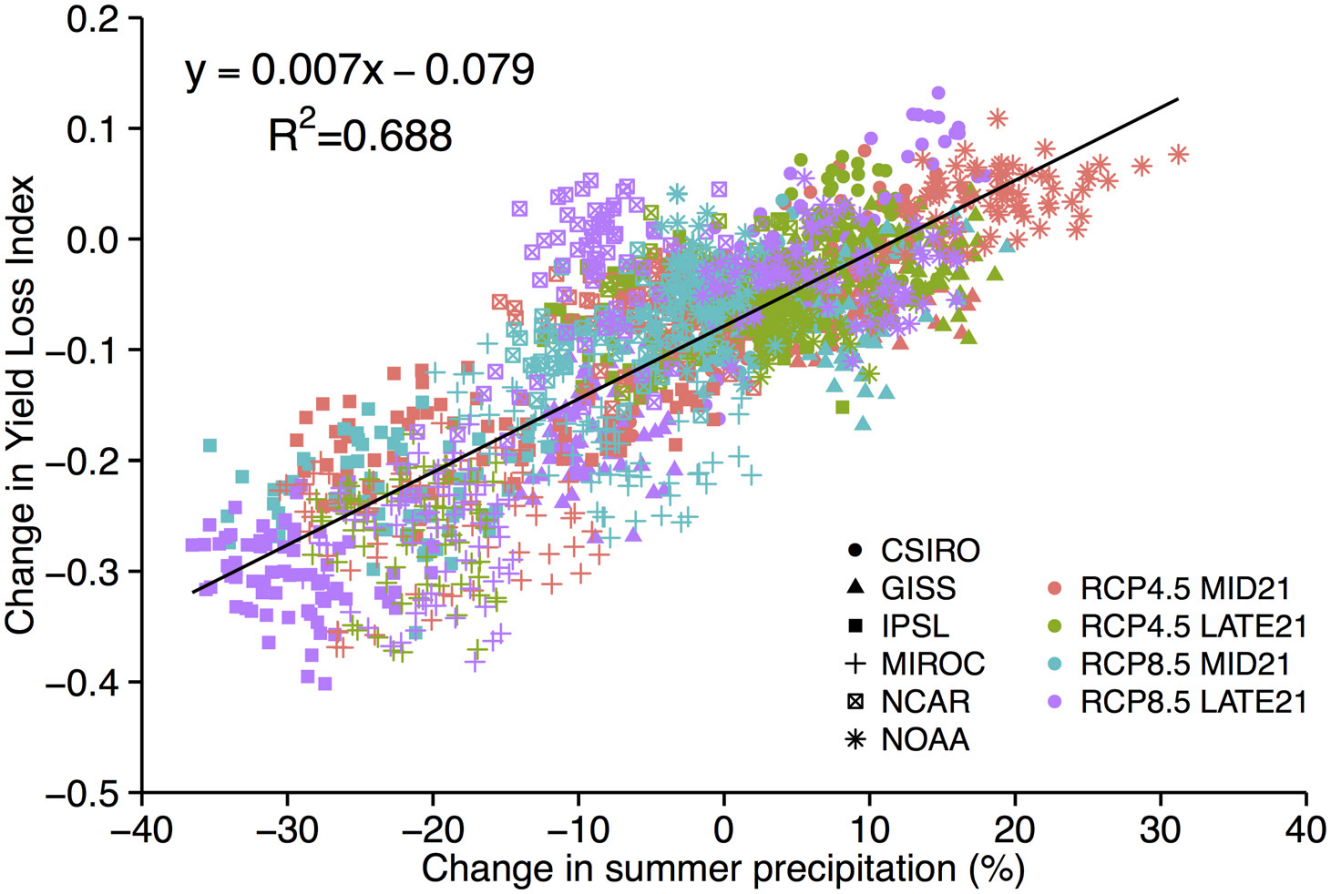
Change in Yield Loss Index vs. change in summer (JJA) precipitation (%) for each GCM and RCP scenario where the value shown is a difference between MID21 or LATE21 and HISTORIC.

The Yield Loss Index (YLI—see Eq 1) is a quantitative measure of the overall impact of precipitation on predicted yield. If yield were not reduced as a result of moisture stress, YLI would be zero (i.e., Yield_NS_ = Yield_D_). This situation would occur with the proper amount and timing of precipitation or irrigation to meet the water needs of maize. Our simulation results show that YLI strengthens (i.e., becomes more negative) under future climate change (Table 4). Note that in the RCP scenarios, both Yield_NS_ and Yield_D_ respond to predicted changes in temperature and other environmental variables such that the only difference between them is that Yield_NS_ does not respond to moisture stress. Only the model runs that have substantial decreases in growing season precipitation, IPSL and MIROC, predict statistically significant differences in YLI, and a third run, GISS, predicts a decrease in YLI only by LATE21 with RCP8.5. In other words, results from four of the six model runs suggest that the predicted losses to Iowa maize yield are primarily a result of higher temperatures, not a predicted increase in moisture stress. The changes in YLI from runs using the IPSL and MIROC data were strongly correlated (R^2^ = 0.69) to the change in summer (June to August) precipitation (Fig 7). The regression indicates that for every 10% decrease in summer precipitation, ~7% of yield would be lost as a result of moisture stress.

**Table 4:** Simulated average maize Yield Loss Index (YLI) averaged across all 72 grid cells in domain from each HISTORIC run driven by the six GCMs and the simulated experimental YLI under both RCP scenarios for the MID21 and LATE21 time periods.

Model	Yield Loss Index				
	HISTORIC	RCP4.5		RCP8.5	
		MID21	LATE21	MID21	LATE21
CSIRO	-0.2045	-0.2560	-0.2023	-0.2544	-0.2082
GISS	-0.1719	-0.2269	-0.1959	-0.2454	**-0.3454**
IPSL	-0.1778	**-0.3562**	-0.2503	**-0.4086**	**-0.4799**
MIROC	-0.1807	**-0.4348**	**-0.4445**	**-0.3598**	**-0.4500**
NCAR	-0.2191	-0.2903	-0.2591	-0.3061	-0.2527
NOAA	-0.1500	-0.1118	-0.2163	-0.1944	-0.1810

Bold values denote values that are significantly different from the HISTORIC value at P<0.01 according to a Student’s t-test. A negative value indicates Yield_D_ < Yield_NS_.

## Discussion

While maize yields in most cropping regions around the world already show a declining trend, yields in Iowa and the USA do not [13], however, all of the model simulations in our present study predict that yields will decline by mid-century, and that these declines will continue with business as usual greenhouse gas emissions and without adaptation to the corresponding changes in climate. Therefore, we need to reduce the uncertainty of maize yield predictions, and evaluate adaptation strategies that farmers can use in the coming decades to maintain maize yields. Results from the DEFAULT simulations predict that Iowa can expect a 6% reduction in maize yield for every 1°C increase in growing season temperature. When any occurrence of moisture stress was removed in the NONSTRESS simulations, simulations predict this reduction to drop to 3%. On the one hand, it is encouraging that yield declines might be halved solely through one adaptation strategy: irrigation. On the other hand, this means that even if Iowa maize were to receive all the water it could use, our model simulations still predict a yield decline of 10–20% from present day average yields.

Our model simulations used the latest climate model output from the CMIP5 suites of models using the updated RCP scenarios to quantify differences in Iowa maize yield projections with and without the response to moisture stress, and our predicted trends in maize yield are comparable to many other studies. The predicted 6% decline in yield per 1°C increase in temperature is close to the 2–5% value found from 1960–2006 for Iowa, Illinois, and Indiana [44], and the 8.3% per 1°C found from 1961–2002 for the globe [14], and also nearly equal to the single season decline of 5% per 1°C increase measured *in situ* at the T-FACE experiment in Illinois [17]. Schlenker and Roberts regressed 1950–2005 USA county-level crop yields with output from four emissions scenarios and a single global climate model and predicted maize yields to decrease 20–30% by 2020–2049 and 45–80% by 2070–2099 [45]. Results from our two RCP scenarios are comparable to their results for MID21 but are a bit lower for LATE21. Our predicted yield decline is also less than the 17% decline per 1°C found by Lobell and Asner [15] for 1982–1998, however, one limitation of their analysis, as well as other statistical analyses, is that correlating yield variability with precipitation variability neglects complex soil-plant-water interactions that make precipitation available to plants through soil moisture [46]. It is possible that our predicted yield declines are less than some statistical models because soil moisture availability has a slower response time than precipitation and can therefore act to buffer relatively short periods of moisture stress from impacting yields.

Ummenhofer et al. used Agro-IBIS, driven with data from the same six GCMs and two RCP scenarios used here, to simulate maize yield change at a single model grid cell in north-central Iowa between 1951–1980 and 2071–2100 [35]. Our goal in the present study was to expand the domain from a single model grid cell to the entire state, to add an additional middle 21^st^ century time period, and to use an HISTORIC baseline period towards the end of the 20^th^ century, 1981–2000. One difference between the present study results and those of Ummenhofer et al. is that while here we find that maize yield is predicted to decline by LATE21 under both RCP4.5 or RCP8.5, with a range of 14–50%, Ummenhofer et al. found a 6% increase in median (from all six models) maize yield under RCP4.5, and a 21% decrease under RCP8.5. In our simulations, only one model predicts yield increases and they occur only for RCP4.5 at MID21 (Fig 4a). Both studies used the same GCMs and RCP scenarios and the same configuration of Agro-IBIS (i.e., a GDD to maturity of 1700), however, they used different observational datasets with which to bias correct the GCM data, they used different domains (one a single grid cell, one the average of 72 grid cells), and most importantly, a different baseline HISTORIC period. The HISTORIC average maize yield in Ummenhofer et al. was ~8 Mg ha^-1^, while in the present study we simulated ~11 Mg ha^-1^. If Ummenhofer et al. predicted a small increase in yield under RCP4.5 by the end of the 21^st^ century, while we predict mainly decreases for both scenarios and both time periods, then these data suggest that we might have passed a peak in maize yield after which yield will only decline.

The only way yields could increase from HISTORIC in the model simulations would be for a combination of increasing temperature and increasing precipitation to result in an environment more favorable for maize growth. While this happened in some simulations in the Ummenhofer et al. study, and yields increased from 8 Mg ha^-1^, our shifting of the HISTORIC time period to produce an average yield of 11 Mg ha^-1^ appears to be near a peak, as yields only decreased from this value in almost all model simulations. There is some other evidence for yields reaching a peak near the turn of the 21^st^ century (see for example [47,48]). Challinor et al. compare findings from a meta-analysis for the Fourth Assessment Report of the Intergovernmental Panel on Climate Change (IPCC AR4; [49]) that suggested that global cereal yields could continue to increase with up to 2°C of warming, before declining, with their new results that showed both increases and decreases for wheat yield and overall greater risk of cereal yield reductions than reported in AR4 [50]. They also suggested that because greater yield declines are predicted for the latter half of the century, that earlier in the century farmer adaptation could mitigate yield loss, but that more transformational strategies will be needed later. Our results support this statement as the strong correlation in simulation results between shortened growing period and increasing temperature in the near future, coupled with a weakening of this relationship later in the century, suggest that yields might be maintained in the near future by alleviating moisture stress and by using warmer season cultivars, but breeding for extreme heat tolerance along with other adaptations might be required to maintain yields by the end of the century.

Ideally, our simulations would have used a variety of statistical downscaling methods to help constrain our range of predicted yield changes resulting from climate change under two RCP scenarios. Here we used one method—the quantile mapping method—to downscale and bias correct monthly GCM projections across Iowa. While we account for some uncertainty in human decision-making by using two of the four RCP scenarios, there is also unknown error in how the model simulates the response of maize to extreme high temperatures and to the variability in extreme weather. We use a statistical weather generator within the Agro-IBIS model with its default parameter settings (developed using 20^th^ century weather data in the USA) in all model simulations to simulate first daily, then hourly variable values rather than using daily data from the GCMs; however, downscaling to monthly rather than daily may not introduce additional error (and may even have less error) [51]. This method may not capture an extremely high temperature value or a brief but soaking rainfall event, either of which could have negative impacts on maize productivity [52]. We also used a single cultivar by simulating 1700 GDD to maturity. Future work should test a range of values to further evaluate impacts of temperature of growing period length. Future work should also include model experiments that test *in situ* measurements of maize response to temperature and drought stress (e.g., with data from T-FACE experiments and, ideally, concurrent with measurements of elevated greenhouse gas response from FACE experiments [53,54]).

## Supporting Information

S1 Fig Change in DEFAULT yield (%) (from top to bottom: RCP4.5 MID21, RCP4.5 LATE21, RCP8.5 MID21, and RCP8.5 LATE21). Hatching indicates a statistically significant (P<0.01) difference according to Student’s t-test.
(TIFF)

S2 Fig Change in NONSTRESS yield (%) (from top to bottom: RCP4.5 MID21, RCP4.5 LATE21, RCP8.5 MID21, and RCP8.5 LATE21). Hatching indicates a statistically significant (P<0.01) difference according to Student’s t-test.
(TIFF)

S3 Fig Change in net solar radiation (%) for each GCM and RCP scenario where the value shown is a percent difference between MID21 or LATE21 and HISTORIC. (TIFF)

S1 Table CMIP5 Models Used in This Study. (DOCX)
