# Supplementary figures and images for: Climate Change and Maize Yield in Iowa
Source: PLoS One. 2016 May 24;11(5):e0156083. doi: 10.1371/journal.pone.0156083 (PMC4878745; doi:10.1371/journal.pone.0156083)

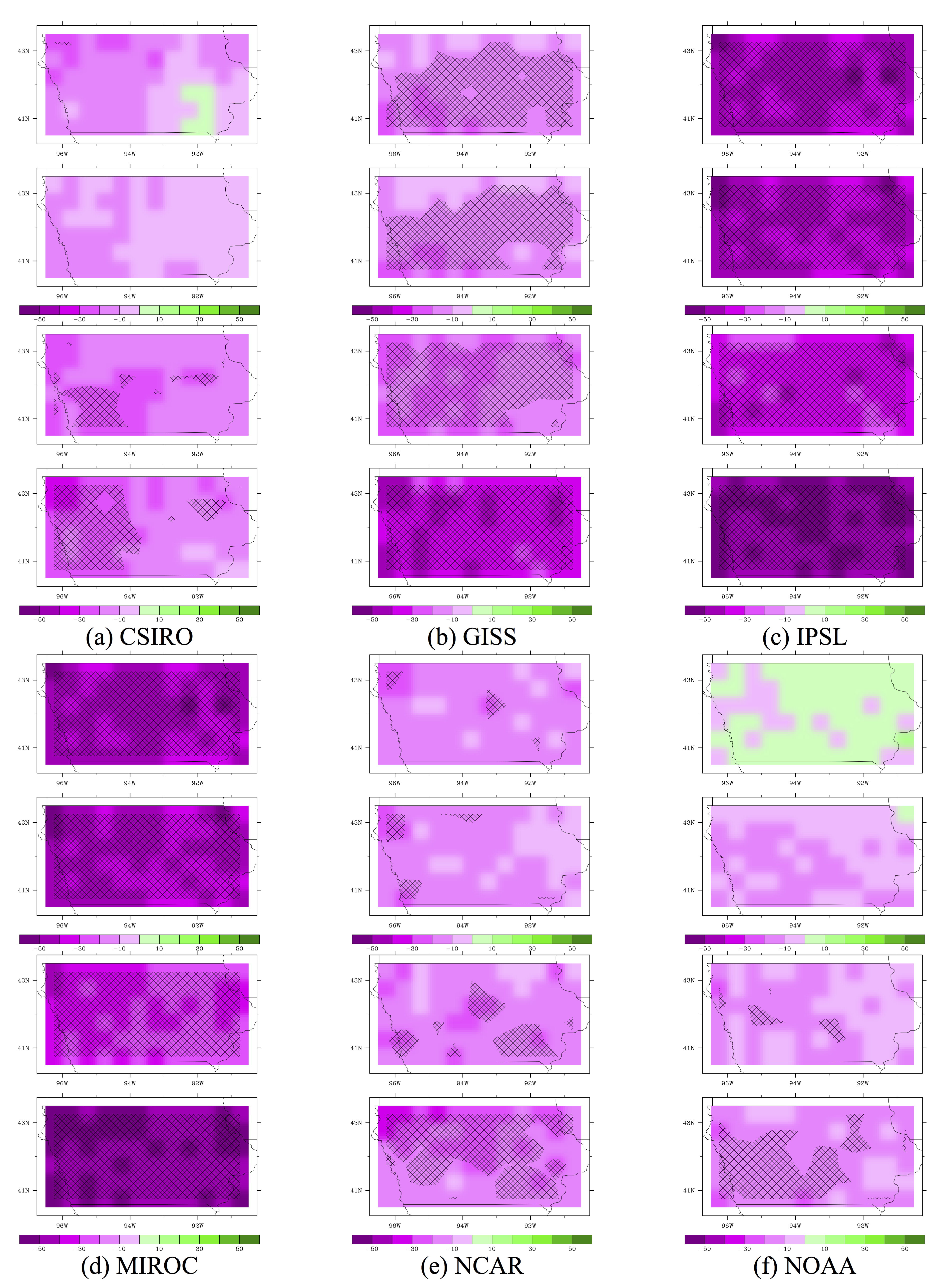

Supplement: S1 Fig — Hatching indicates a statistically significant (P<0.01) difference according to Student’s t-test. (TIFF) [file pone.0156083.s001.tiff]

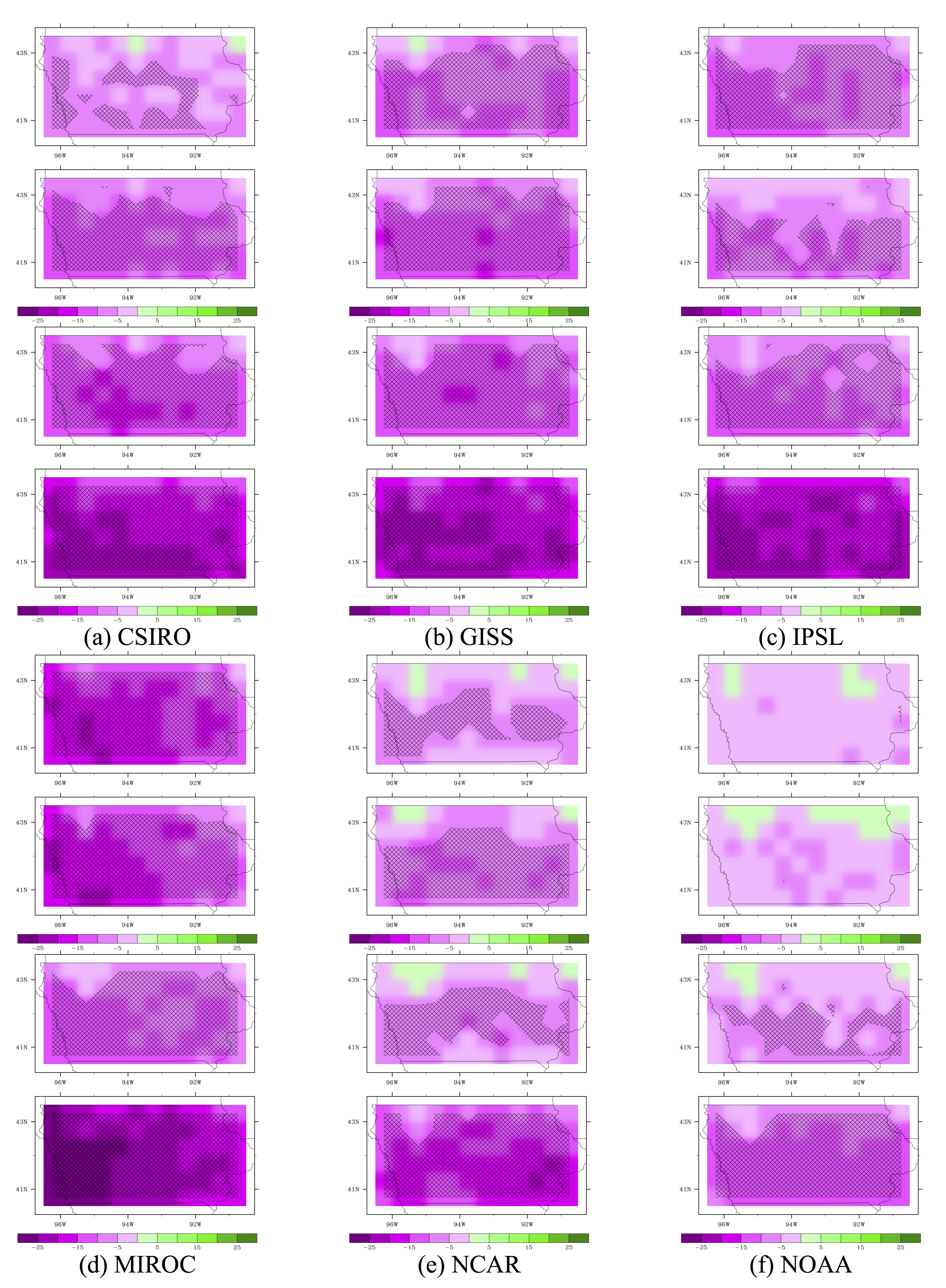

Supplement: S2 Fig — Hatching indicates a statistically significant (P<0.01) difference according to Student’s t-test. (TIFF) [file pone.0156083.s002.tiff]

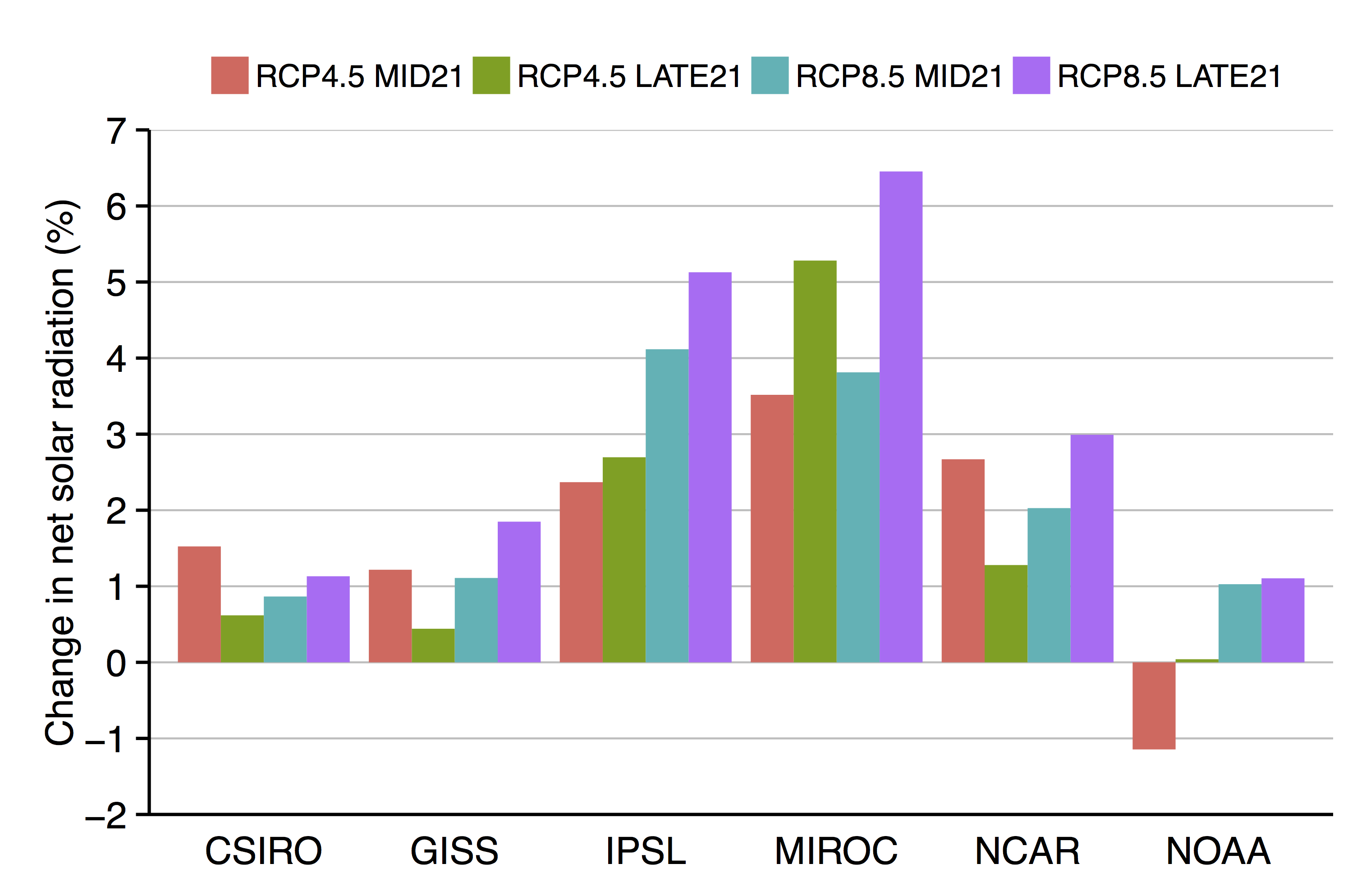

Supplement: S3 Fig — (TIFF) [file pone.0156083.s003.tiff]
