# Supplementary material for: Climate Change and Maize Yield in Iowa
Source: PLoS One. 2016 May 24;11(5):e0156083. doi: 10.1371/journal.pone.0156083 (PMC4878745; doi:10.1371/journal.pone.0156083)
Supplement: S1 Table — (DOCX) [file pone.0156083.s004.docx]

**S1 Table. CMIP5 Models Used in This Study.**

| Model | Institution | Resolution  (degrees) |
| --- | --- | --- |
| CSIRO-Mk3.6.0 | Commonwealth Scientific and Industrial Research Organisation-  Queensland Climate Change Centre of Excellence  (CSIRO-QCCCE; Australia) | 1.875 x 1.875 |
| GISS-E2-R | National Aeronautics and Space Administration  Goddard Institute for Space Sciences  (NASA GISS; USA) | 2 x 2.5 |
| IPSL-CM5a-MR | Institut Pierre-Simon LaPlace  (IPSL; France) | 1.25 x 2.5 |
| MIROC5 | Model for Interdisciplinary Research on Climate  (MIROC; Japan) | 2.8 x 2.8 |
| CCSM4 | National Center for Atmospheric Research  (NCAR; USA) | 0.9 x 1.25 |
| GFDL-ESM2M | National Oceanic and Atmospheric Administration-  Geophysical Fluid Dynamics Laboratory  (NOAA-GFDL; USA) | 2 x 2.5 |
